# 972 Evaluating Cool Running Water Implementation for Acute Burn Injuries in the Emergency Department

**DOI:** 10.1093/jbcr/iraf019.503

**Published:** 2025-04-01

**Authors:** Maleea Holbert, Tina Palmieri, John Rose, Nathan Kuppermann, Jeremy Veldstra, Katrina Falwell, Bronwyn Griffin

**Affiliations:** Queensland Children’s Hospital and Griffith University; University of California, Davis and Shriners Children’s; University of California Davis; Children’s National Hospital and George Washington University School of Medicine; University of California Davis Medical Center; University of California Davis Health; Griffith University

## Abstract

**Introduction:**

Application of 20 minutes of cool running water (20CRW) to a burn within the first three hours post-injury significantly improves patient outcomes and is first-line treatment in Australia, New Zealand, Europe, and the United Kingdom. We assessed the implementation of this burn first aid treatment within the emergency department (ED) of a major burn referral hospital in December 2023.

**Methods:**

ED clinicians (medical, nursing, and technicians) completed online questionnaires after providing 20CRW to patients who had suffered acute burn injuries. Questionnaires assessed issues specific to each patient’s burn injuries and disposition and were aimed to capture clinicians’ experience of administering 20CRW, challenges faced, additional resources and adaptions required, and their overall satisfaction. Free text data were coded using inductive content analysis. Consensus meetings with relevant clinical stakeholders were then conducted to adapt implementation strategies and resources based on data reported in questionnaires.

**Results:**

A total of 65 ED clinicians completed the questionnaire, resulting in a response rate of 71%. Over 34% of respondents reported that 20CRW was very easy to administer; 21% indicated it was easy to administer; 21% were neutral; 15% reported it was difficult; and 9% reported it was very difficult. Free text themes identified several patient challenges to providing 20CRW, including non-compliance, combativeness, non-ambulatory status, pediatrics, and burns affecting multiple anatomical regions. Operational challenges reported included limited access to sinks and showers, time constraints, and staffing shortages. Despite these barriers, clinicians highlighted the effectiveness of 20CRW in alleviating patient pain, as reflected in 14% of responses. One participant stated, “It’s super helpful and patients say it helps with pain. Patients are willing to do it because it feels good. I was initially nervous patients would resist, but that’s not the case.” Resource requests following implementation included improving the documentation of 20CRW application in the electronic health record; continued education on inclusion and exclusion criteria for 20CRW; reliable portable showers for bedside irrigation; waterproof footwear; and equipment for positioning patients under the shower.

**Conclusions:**

Clinicians found 20CRW easy to administer; however, there are notable challenges, particularly with specific patient groups and logistical constraints. Despite these challenges, clinicians consistently recognize the effectiveness of 20CRW in pain alleviation. Continued monitoring and iterative improvements will be needed to address identified challenges and ensure long-term sustainment of this burn first aid treatment.

**Applicability of Research to Practice:**

This study identifies barriers to the implementation of 20CRW in emergency departments as well as solutions to overcome those barriers.

**Funding for the Study:**

This research received competitive government grant funding, awarded to the Principal Investigator.

